# Country data on AMR in Russia in the context of community-acquired respiratory tract infections: links between antibiotic susceptibility, local and international antibiotic prescribing guidelines, access to medicine and clinical outcome

**DOI:** 10.1093/jac/dkac218

**Published:** 2022-09-06

**Authors:** Didem Torumkuney, Roman Kozlov, Sergey Sidorenko, Praveen Kamble, Margarita Lezhnina, Aleksandr Galushkin, Subhashri Kundu

**Affiliations:** GlaxoSmithKline, 980 Great West Road, Brentford, Middlesex TW8 9GS, UK; Smolensk State Medical University (SSMU), Krupskaya Str. 28 Smolensk 214019, Russia; Pediatric Research and Clinical Centre for Infectious Diseases, Professor Popov Str. 9, Russia; North Western State Medical University named after I. I. Mechnikov, Kirochnaya Str. 41, St Petersburg 195067, Russia; GlaxoSmithKline, 252, Dr Annie Besant Road, Worli, 400030, Mumbai, India; GlaxoSmithKline Trading, Leningrad’s prospect, 37A, bld. 4, Arcus III, 125167 Moscow, Russia; GlaxoSmithKline Trading, Leningrad’s prospect, 37A, bld. 4, Arcus III, 125167 Moscow, Russia; GlaxoSmithKline, 23 Rochester Park, 139234, Singapore

## Abstract

**Background:**

Antimicrobial reistance (AMR) is one of the biggest threats to global public health. Selection of resistant bacteria is driven by inappropriate use of antibiotics, amongst other factors. COVID-19 may have exacerbated AMR due to unnecessary antibiotic prescribing. Country-level knowledge is needed to understand options for action.

**Objectives:**

To review AMR in Russia and any initiatives addressing it. Identifying any areas where more information is required will provide a call to action to minimize any further rise in AMR within Russia and to improve patient outcomes.

**Methods:**

National AMR initiatives, antibiotic use and prescribing, and availability of susceptibility data, in particular for the key community-acquired respiratory tract infection (CA-RTI) pathogens *Streptococcus pneumoniae* and *Haemophilus influenzae*, were identified. National and international antibiotic prescribing guidelines commonly used locally for specific CA-RTIs (community-acquired pneumonia, acute otitis media and acute bacterial rhinosinusitis) were also reviewed, plus local antibiotic availability. Insights from both a local clinician and a local clinical microbiologist were sought to contextualize this information.

**Conclusions:**

Russia launched a national strategy in 2017 to prevent the spread of AMR and the WHO reports that as of 2020–21, it is being implemented and actively monitored. Reports suggest outpatient antibiotic use of antibiotics is high and that non-prescription access and self-medication are very common. Antibiotic susceptibility studies in Russia include PeHASus, a multicentre epidemiological study focusing on susceptibilities of community-acquired respiratory pathogens and international studies such as Survey of Antibiotic Resistance (SOAR), Antimicrobial Testing Leadership and Surveillance (ATLAS) and SENTRY Antimicrobial Surveillance Program. International guidelines are used to support the development of local guidelines in Russia, and for the common CA-RTIs Russian clinicians use of several country-specific local antibiotic prescribing guidelines. A standardized inclusive approach in developing local guidelines, using up-to-date surveillance data of isolates from community-acquired infections in Russia, could make guideline use more locally relevant for clinicians. This would pave the way for a higher level of appropriate antibiotic prescribing and improved adherence. This would, in turn, potentially limit AMR development and improve patient outcomes.

## Introduction

Antimicrobial resistance (AMR) is one of the biggest threats to public health throughout the world^1^ as described in the introductory paper of this Supplement.^2^ The WHO states that ‘the world urgently needs to change the way it prescribes and uses antibiotics. Even if new medicines are developed, without behaviour change, antibiotic resistance will remain a major threat’.^3^ The first paper in this Supplement included details about the multiple factors which can drive a rise in AMR along with the global initiatives that are in place to address this threat.^2^ Each country and/or region must also play their part through local initiatives.

In order to identify how AMR can be addressed in Russia in the future, it is necessary to review what is happening now. In this paper, we present the current situation in Russia, determined by using published information (from searching PubMed, Google Scholar and other internet platforms) to ascertain any national initiatives to address AMR in Russia, antibiotic use and prescribing, and availability of susceptibility data, in particular for the key community-acquired respiratory tract infection (CA-RTI) pathogens *Streptococcus pneumoniae* and *Haemophilus influenzae*. National and international antibiotic prescribing guidelines for CA-RTIs, specifically community-acquired pneumonia (CAP), acute otitis media (AOM) and acute bacterial rhinosinusitis (ABRS), commonly used by healthcare professionals (HCPs) in Russia were also reviewed, along with how these link to local antibiotic availability. Insights from a clinician and a clinical microbiologist were sought to contextualize this information. In addition, we aimed to identify areas where more information is required and present a call to action to improve clinical outcomes for patients and to minimize further rises in AMR within Russia.

## Action Plans

Following the formulation by the World Health Assembly in 2015 of a Global Action Plan (GAP) for AMR,^4^ many countries began to develop their own National Action Plan (NAP). Following global movements to tackle AMR, Russia launched a national strategy in 2017 to prevent the spread of AMR. In a similar way to the GAP on AMR, the Russian plan emphasizes the importance of addressing the misuse of antibiotics in healthcare practices and overprescribing of antibiotics by HCPs and recognizes the need for behavioural changes amongst antibiotic users and prescribers.^5,6^ Current NAP status, as reported by the WHO for 2020–21, shows that for Russia the AMR NAP is being implemented and actively monitored through a monitoring and evaluation framework.^7^

## Antibiotic usage

The consumption of antibiotics in Russia is reported as 14.82 DDD per 1000 people per day, lower than the median consumption of antibiotics for the WHO European region (including 45 countries and Kosovo) which is 17.9 DDD per 1000 people per day.^8^ Other reports suggest that the outpatient use of antibiotics is high and that non-prescription access and self-medication of antibiotics are also very common in Russia.^9^

A survey carried out in 2015 in Saint Petersburg found that self-prescription of antibiotics, usually for respiratory tract infections (RTIs), was common amongst pharmacists, raising concerns about the inappropriate use of antibiotics in infections such as coughs and colds.^9^ A prohibition on the over-the-counter (OTC) sale of antibiotics without a prescription has been in place in Russia since 2006, but no mechanisms existed for its enforcement. In 2017, the federal government introduced several regulation changes relating to drug circulation, and the prescription of antibiotics by doctors became obligatory. Surprise inspections of pharmacies were implemented to enforce control of OTC sales of medications, including antibiotics. Pharmacists were also obliged to collect and file patient prescriptions for inspection purposes.^5^

## Surveillance

### National surveillance studies

**Figure 1.**
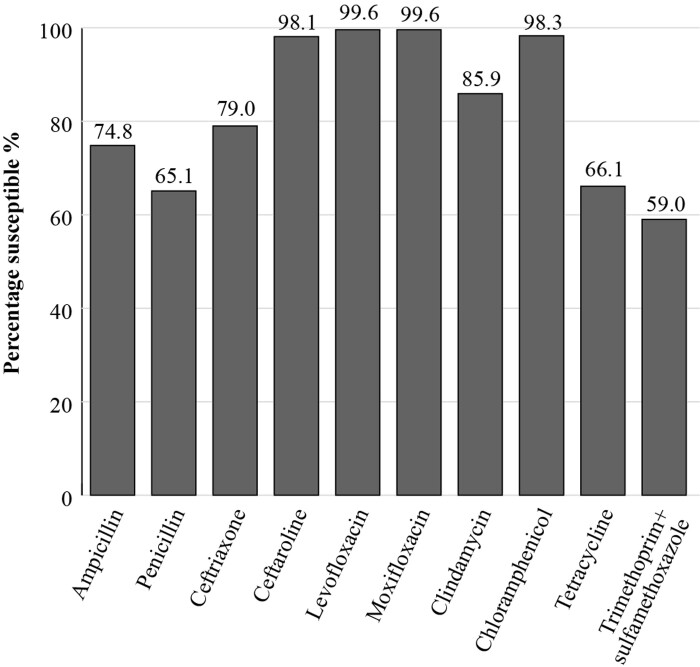
Percentage susceptibility rates based on EUCAST breakpoints for antibiotics against *S. pneumoniae* isolates (*n *= 519) collected as part of the PeHASus surveillance study in Russia in 2014–17.

**Figure 2.**
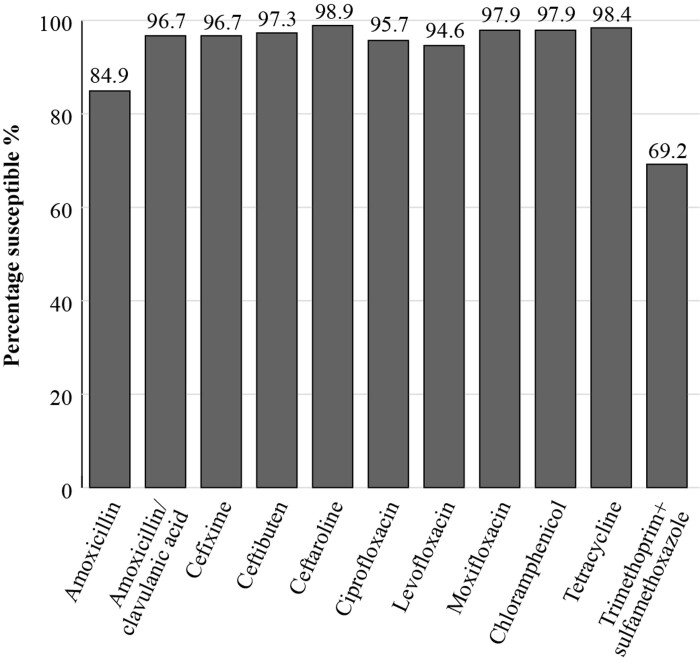
Percentage susceptibility rates based on EUCAST breakpoints for antibiotics against *H. influenzae* isolates (*n *= 185) collected as part of the PeHASus surveillance study in Russia 2014–17.

PeHASus is a Russian multicentre epidemiological study focusing on antibiotic susceptibilities of community-acquired respiratory pathogens. The susceptibilities, applying EUCAST breakpoints, of 519 *S. pneumoniae* isolates collected between 2014 and 2017 are shown in Figure 1. The prevalence of susceptibility to penicillin was 65.1%, and to trimethoprim/sulfamethoxazole it was 59%.^10^ The susceptibility of 185 isolates of *H. influenzae* collected from centres between 2014 and 2017 was also investigated in this study. Isolates mostly showed a high susceptibility to all antibiotics tested (excluding trimethoprim/sulfamethoxazole) including amoxicillin/clavulanic acid (96.7% susceptibility by EUCAST breakpoints). The prevalence of susceptibility to amoxicillin alone was 84.9%. These data demonstrate the importance of using β-lactam+β-lactamase inhibitor combinations to treat *H. influenzae* infections (Figure 2).^11^

AMRmap is a web resource that provides access to the Russian national AMR surveillance system.^12^ The surveillance system includes information obtained from centralized testing of microbial isolates received from participating laboratories across Russia. The platform currently comprises the susceptibility data of more than 40 000 clinical isolates.^12^

### Global surveillance studies

#### SOAR

Several ongoing global surveillance studies provide antibiotic susceptibility data in Russia. An early programme was GlaxoSmithKline’s Survey of Antibiotic Resistance (SOAR), a multinational antibiotic surveillance study, ongoing in an expanding range of countries since 2002. The study aims to collect and make available in published, peer-reviewed papers, antibiotic susceptibility data, specifically for *S. pneumoniae* and *H. influenzae*, the most commonly isolated respiratory pathogens in the community.^13^ Key features of the SOAR study are that it focuses on only these pathogens, and that identification and susceptibility testing are performed in an independent centralized laboratory using standardized methodology from CLSI, allowing comparisons to be made between countries/regions and for the identification of trends over time. SOAR data is analysed based on three different breakpoints: CLSI, EUCAST dose-specific and PK/PD breakpoints.

Clinical breakpoints are cut-off MIC values used to classify microorganisms into the clinical categories susceptible (S), intermediate (I) and resistant (R) to assist in the prediction of the clinical success or failure of a specific antibiotic.^14^ Two international organizations define breakpoint values: CLSI and EUCAST. Due to variation in criteria for their definition, there are some differences between CLSI and EUCAST in the clinical breakpoint values for certain bacteria for some antibiotics and this can impact susceptibility interpretation of clinical isolates.^15^ EUCAST breakpoints are dose-specific and use the EMA-approved doses that are included in the Summary of Product Characteristics of an antibiotic. This means that by application of breakpoints for higher doses, the effect of using a raised dose on the clinical efficacy of a particular antibiotic can be predicted. Currently, both CLSI and EUCAST breakpoints are used in clinical microbiology laboratories in Russia. The international application of the EUCAST breakpoints is expanding^16^ and the EUCAST dose-specific breakpoints can also be used retrospectively to calculate the susceptibility of previously collected isolates to show the susceptibility levels that would have been achieved at higher doses.

Use of the EUCAST dose-specific breakpoints shows the effect of increasing the antibiotic dose on the susceptibility of a pathogen, providing additional information so the prescriber can decide if a higher dose would be of benefit. For example, *S. pneumoniae*, the most isolated respiratory pathogen^17,18^ for infections such as CAP, AOM and ABRS, has over time become less susceptible to amoxicillin/clavulanic acid in some countries since the MIC of some isolates has increased. When treating infections, it is important to be able to eradicate bacterial pathogens with raised MICs to optimize clinical outcomes while at the same time minimizing the risk of selecting variants with even-higher MICs. This is possible because β-lactams, unlike many other antibiotics, have time-dependent killing properties. Their efficacy depends on the amount of time the drug concentration is present at the site of action. Although increasing the concentration at the infection site over a particular concentration will not have any effect on the efficacy, the use of higher doses and/or more frequent dosing allows for successful eradication of infections caused by pathogens with higher MICs because the time above the MIC is increased.^19^

**Figure 3.**
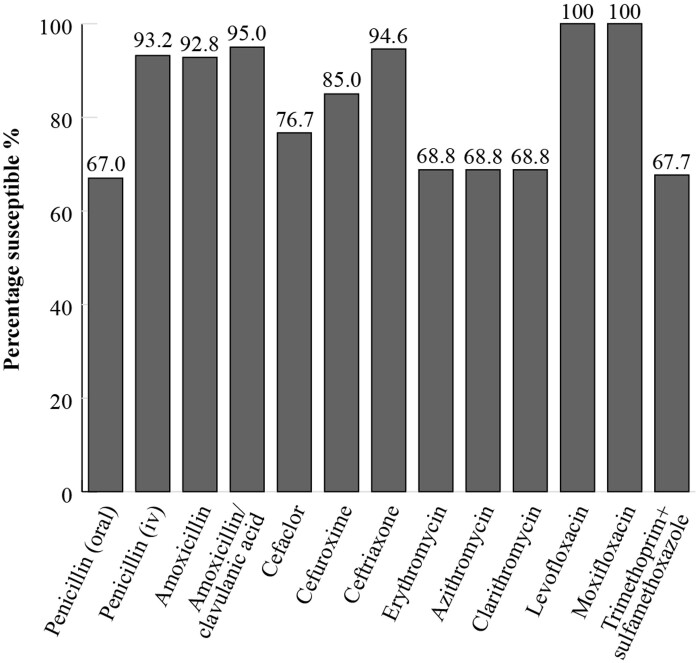
Percentage susceptibility rates based on CLSI breakpoints for antibiotics against *S. pneumoniae* isolates (*n *= 279) collected as part of the SOAR study in Russia in 2014–16.

In the SOAR study in Russia, isolates were collected at three sites between 2014 and 2016 from outpatients with confirmed CA-RTIs. When applying CLSI breakpoints, 95% of *S. pneumoniae* isolates (*n *= 279) were susceptible to amoxicillin/clavulanic acid and 92.8% to amoxicillin (Figure 3). For the macrolides, erythromycin, azithromycin and clarithromycin, 68.8% of isolates were susceptible. For the fluoroquinolones, levofloxacin and moxifloxacin, *S. pneumoniae* susceptibility was 100% (Figure 3).^13^

**Figure 4.**
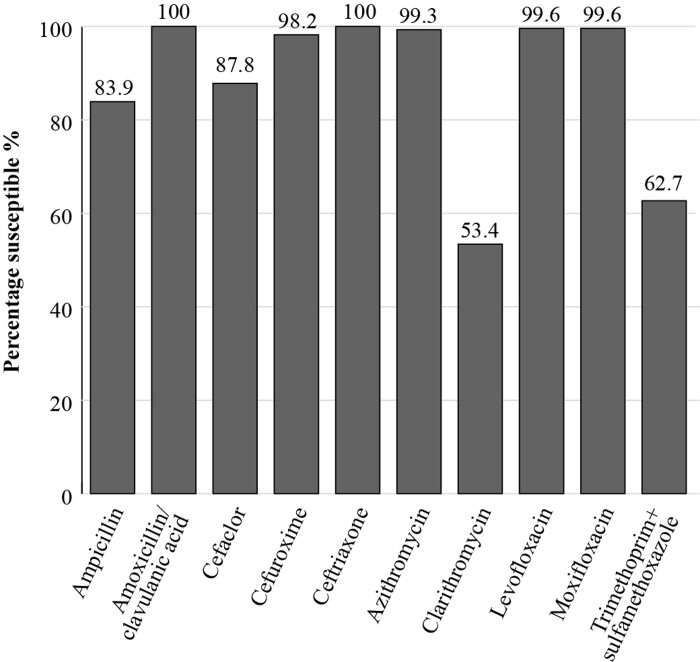
Percentage susceptibility rates based on CLSI breakpoints for antibiotics against *H. influenzae* isolates (*n *= 279) collected as part of the SOAR study in Russia in 2014–16.

All *H. influenzae* isolates (*n *= 279) from Russia demonstrated a high susceptibility (100%) to amoxicillin/clavulanic acid using CLSI criteria (Figure 4). Susceptibility to ampicillin was 83.9% by CLSI criteria, which reflects the prevalence of β-lactamase-positive isolates. Clarithromycin and trimethoprim/sulfamethoxazole showed low susceptibility using CLSI (53.4% and 62.7%, respectively).^13^

#### ATLAS

**Figure 5.**
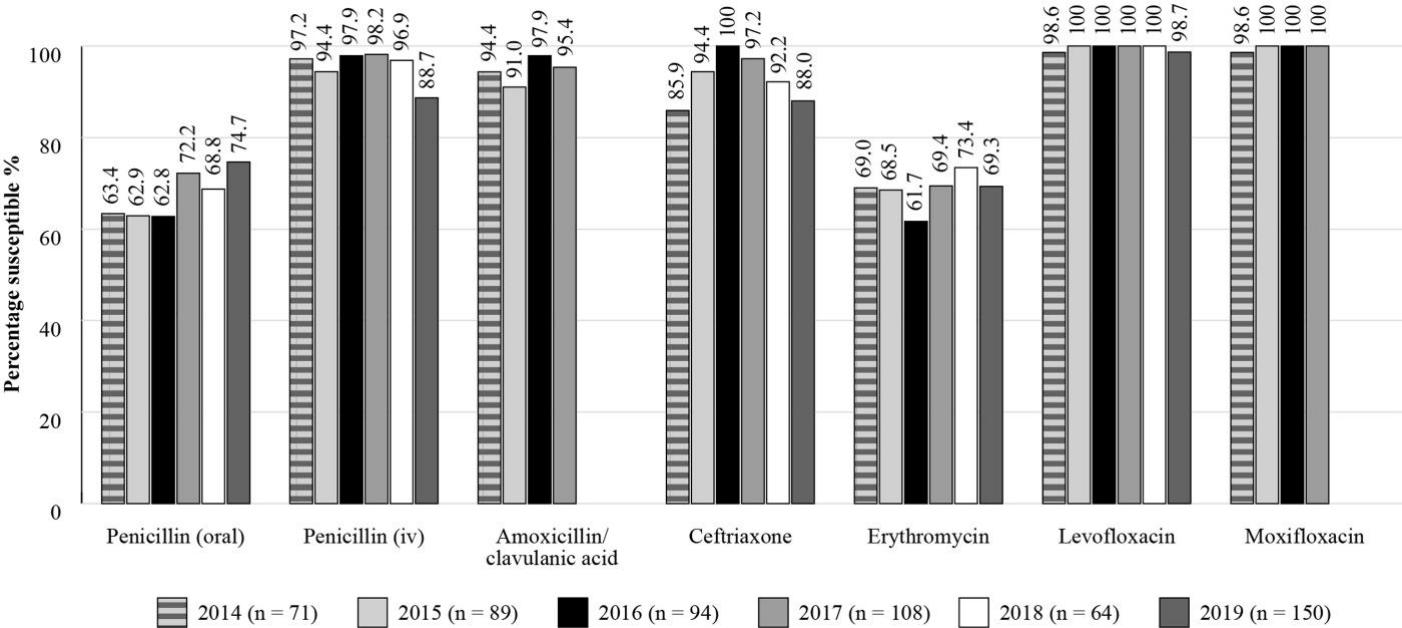
Percentage susceptibility rates based on CLSI breakpoints for antibiotics against *S. pneumoniae* isolates collected in Russia as part of the ATLAS study in 2014–19. Data access date 22 November 2021.

The Antimicrobial Testing Leadership and Surveillance (ATLAS) database is a global AMR surveillance programme that is fully searchable, available for general access and covers susceptibilities of a range of bacterial and fungal pathogens to a bank of antimicrobials.^20^ ATLAS data is analysed based on CLSI/EUCAST breakpoints. Susceptibility data for Russia is available for *S. pneumoniae* isolates from between 2014 and 2019 (*n *= 71 in 2014; *n* = 89 in 2015; *n *= 94 in 2016; *n *= 108 in 2017; *n *= 64 in 2018; *n *= 150 in 2019) (Figure 5). The susceptibility of *S. pneumoniae* isolates to amoxicillin/clavulanic acid ranged from 91.0% to 97.9% during this period (based on CLSI criteria). Susceptibility of *S. pneumoniae* isolates was lower to the macrolide erythromycin (based on CLSI guidelines, percentage susceptibility to erythromycin reflects the percentage susceptibility to azithromycin and clarithromycin) being between 61.7% and 73.4%, but was high for the fluoroquinolones, levofloxacin and moxifloxacin (98.6%–100%).

#### SENTRY

The SENTRY Antimicrobial Surveillance Program, another long running study, was established in 1997. SENTRY monitors worldwide pathogens and the changes in resistance patterns over time through centralized testing, utilizing reference susceptibility methods.^21^

**Figure 6.**
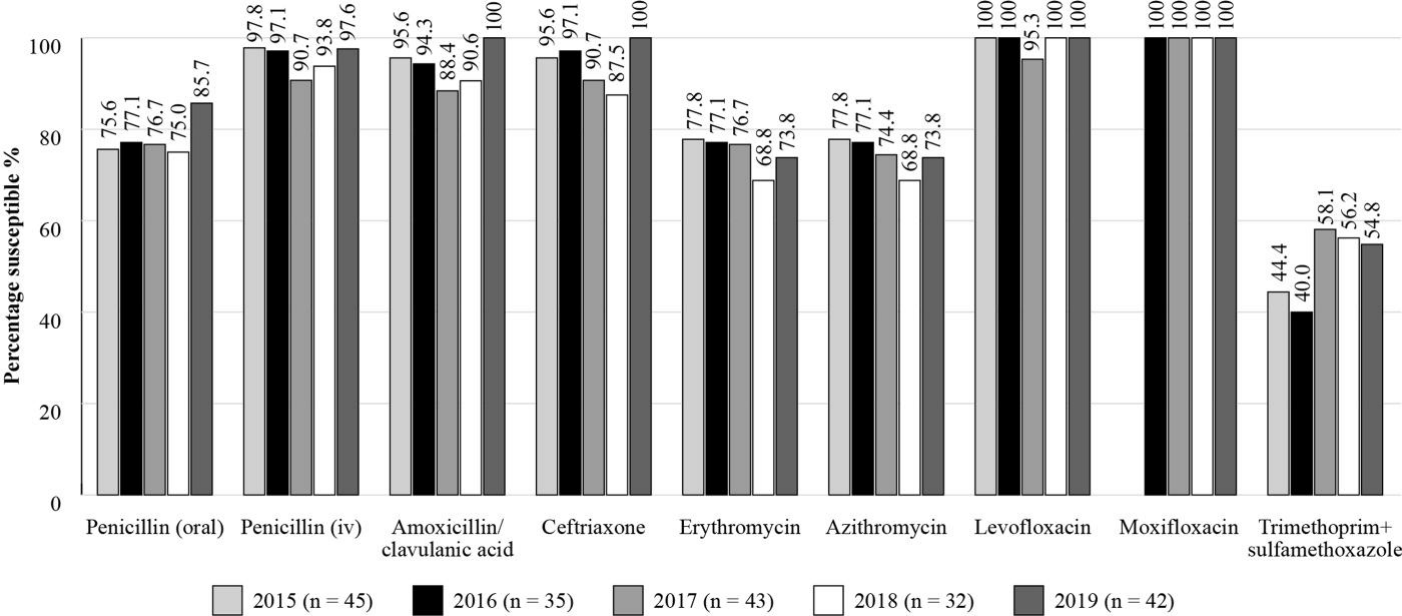
Percentage susceptibility rates based on CLSI breakpoints for antibiotics against *S. pneumoniae* isolates collected in Russia as part of the SENTRY study in 2015–19. Data access date 22 November 2021.

**Figure 7.**
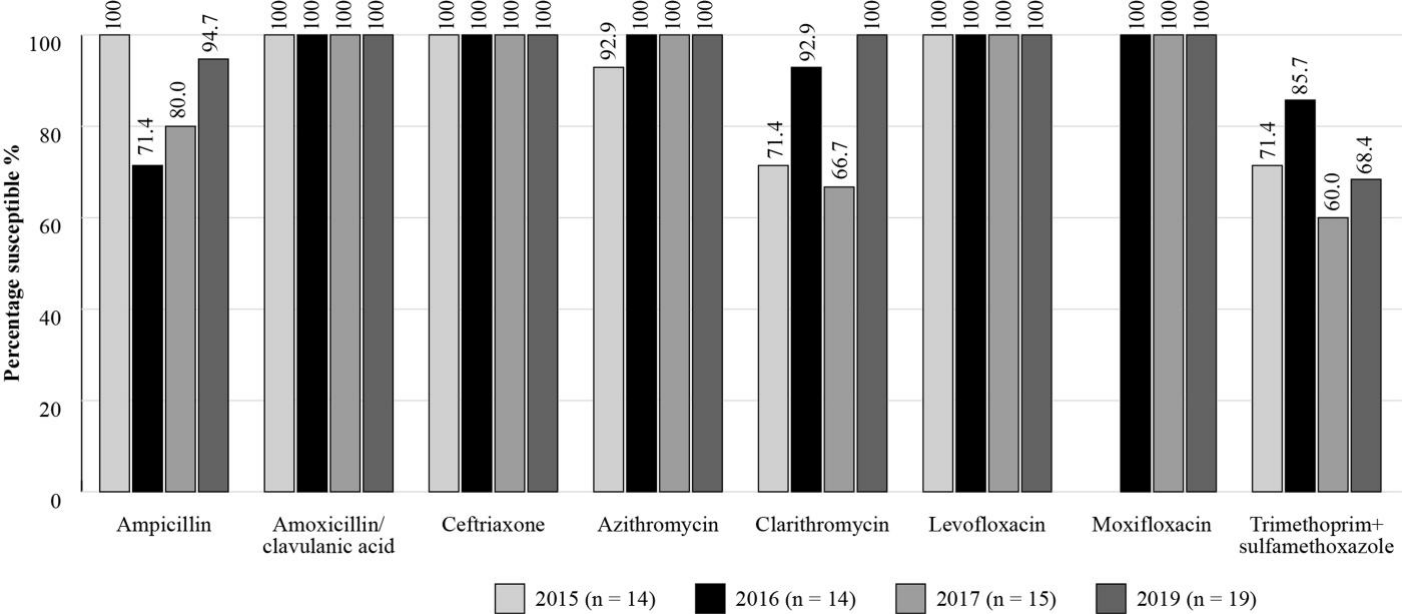
Percentage susceptibility rates based on CLSI breakpoints for antibiotics against *H. influenzae* isolates collected in Russia as part of the SENTRY study in 2015–17 and 2019 (2018 data are omitted owing to low isolate numbers). Data access date 22 November 2021.

Susceptibility data for Russia are available for *S. pneumoniae* (*n *= 45 in 2015; *n *= 35 in 2016; *n *= 43 in 2017; *n *= 32 in 2018; *n* = 42 in 2019) and *H. influenzae* (*n *= 14 in 2015; *n *= 14 in 2016; *n *= 15 in 2017 and *n *= 19 in 2019; 2018 omitted due to low isolate numbers) isolates between 2015 and 2019 (Figures 6 and 7). Results corroborate those seen with the other studies, with a high susceptibility of *S. pneumoniae* isolates to amoxicillin/clavulanic acid, penicillin IV, ceftriaxone, levofloxacin and moxifloxacin during this period, using CLSI criteria.

#### GLASS

In 2015, the WHO launched the Global Antimicrobial Resistance and Use Surveillance System (GLASS). GLASS is the first global system to collect national AMR data for selected bacterial pathogens that cause common infections. The aim is to monitor the prevalence of AMR among major pathogens in clinical settings to provide the supporting data required to ensure that countries can design cost-effective, evidence-based AMR response strategies.^22^ During the first four years, 91 countries/territories have enrolled in GLASS and data for over two million patients from 66 countries are included.^23^ Pathogens currently included in GLASS-AMR are: *Acinetobacter* spp., *Escherichia coli*, *Klebsiella pneumoniae*, *Neisseria gonorrhoeae*, *Salmonella* spp., *Shigella* spp., *Staphylococcus aureus*, and *S. pneumoniae* and a new and important component is the inclusion of antimicrobial consumption (AMC) surveillance at the national level.^24^ GLASS data is analysed based on CLSI and EUCAST breakpoints. Russia is participating in the GLASS initiative and according to the early implementation report for 2020, the National AMR Surveillance network comprises about 50 laboratories that provide microbiological services for more than 65 medical facilities. The central laboratory of the Institute of Antimicrobial Chemotherapy annually collects clinical isolates from each participating medical centre.^25^

## Disease Management Guidelines

Most guidelines suggest a first-line antibiotic or antibiotics along with alternatives and then a second-line antibiotic or antibiotics, also with alternatives. The first-line antibiotic is the recommended initial choice that should be prescribed by the clinician following diagnosis of the infection, supported by the criteria defined by the organization or committee; alternatives may be provided for use in particular circumstances. For example, if the first-line antibiotic is a penicillin then alternative suggestions would be for use in the case of penicillin allergy. The second-line antibiotic is for use if the first-line antibiotic does not achieve the anticipated outcome, and again alternatives may be included for specific circumstances.

For the management of the common CA-RTIs, Russian clinicians make use of several country-specific local antibiotic prescribing guidelines, examples of which are shown in Table 1. International guidelines are used to support the development of local guidelines in Russia.

**Table 1.** Examples of national antibiotic prescribing guidelines referred to by physicians in Russia for the management of community-acquired respiratory tract infections

National guidelines
Community-acquired pneumonia in children, 2015^26^
Acute otitis media, 2021^27^
Acute bacterial rhinosinusitis, 2021^28^
Community-acquired pneumonia in adults, 2021^29^

### National antibiotic prescribing guidelines

Local Russian guidelines are approved by the Russian Ministry of Health and are available for the management of CAP, AOM and ABRS. For CAP in children aged 3 months to 5 years, amoxicillin is the first-line treatment and amoxicillin/clavulanic acid is an alternative option (with amoxicillin/sulbactam, cefuroxime axetil and cefixime) where there is mixed infection or suspicion of it in a patient who has received β-lactam antibiotics in the last 3 months, or if there is a risk of *H. influenzae* β-lactamases. A higher dose regimen (90 mg/kg/day twice daily) is recommended where there is the risk, or presence, of penicillin-resistant *S. pneumoniae*.^26^ In adults with CAP, amoxicillin/clavulanic acid (500 mg/125 mg three times daily or 875 mg/125 mg twice daily) or ampicillin/sulbactam are recommended as first-line for non-severe CAP patients with co-morbidities, in those who have taken antibiotics for more than 2 days in the previous 3 months, and in those who have other risk factors.^29^ For ABRS in both adults and children, the guideline recommends, amoxicillin as the first choice empirical therapy and amoxicillin/clavulanic acid as an alternative option. Amoxicillin/clavulanic acid is considered as a starting empirical therapy where there is a risk of antibiotic resistance or failure of initial therapy with amoxicillin, in patients with comorbidities, immunosuppressive conditions or aged >65 years.^28^ In adults and children with AOM, amoxicillin/clavulanic acid is recommended where there are risk factors for the presence of resistant isolates of *H. influenzae* or *Moraxella catarrhalis* and failure of initial treatment with a 3 day course of amoxicillin, and if there is combination of symptoms of otitis media and conjunctivitis, if antibiotic therapy was carried out during the previous month and in children who are often ill or attend kindergartens. Intravenous amoxicillin/clavulanic acid is also proposed where parenteral therapy is necessary.^27^

## Antibiotic availability

Access to antibiotics may be an issue for patients in low- and middle-income countries due to cost and insufficient government expenditure or support in this area. Drug supply chains may also contribute to the problem. Limited access to the most appropriate antibiotic to treat a specific infection may result in raised mortality from treatable bacterial infections, and the use of suboptimal amounts of antibiotic facilitates resistance development and allows resistant strains to persist.^30,31^

Using amoxicillin/clavulanic acid as an example, within Russia, a range of formulations currently available are mentioned as first- or second-line recommendations by the National RTI management guidelines. For example, in the CAP guideline for outpatients, the following regimens of amoxicillin/clavulanic acid: 500 mg every 8 h or 875 mg every 12 h are recommended as the first-line treatment for non-severe CAP in outpatients with comorbidities, other risk factors or who have taken antibiotics for more than 2 days in the last 3 months.^29^ In children with CAP, 45–50 mg/kg/day amoxicillin/clavulanic acid (as amoxicillin component) is recommended in the presence or suspicion of mixed infections, in those receiving β-lactam antibiotics in the last 3 months, or where β-lactamase-producing *H. influenzae* are present or suspected. The ‘high dose’ regimen, 90 mg/kg/day (as amoxicillin component) in two divided doses is recommended where there is a risk or presence of *S. pneumoniae* resistance to penicillin.^26^ In ABRS, amoxicillin/clavulanic acid is recommended as an alternative empirical starting therapy where there is a risk of β-lactamase-producing pathogens or in patients with comorbidities or immunosuppression or patients >65 years old or failure of initial therapy, in the following regimens: 625 mg three times a day or 1000 mg twice a day. In children, amoxicillin/clavulanic acid 45–60 mg/kg/day in 2–3 doses is an alternative for empirical therapy and the ‘high dose’ amoxicillin regimen 90 mg/kg/day is recommended where there is a risk of antibiotic resistance.^28^ In AOM in children, the guidelines recommend the standard dose regimens (formulations 7:1, ratio amoxicillin:clavulanic acid) of amoxicillin/clavulanic acid 45–60 mg/kg/day given in 2–3 doses (based on amoxicillin dose) for use where there are risk factors for resistant isolates of *H. influenzae* and *M. catarrhalis* and failure of initial antibiotic treatment with amoxicillin. The ‘high dose’ (14:1 ratio) regimen of 90 mg/kg/day (amoxicillin component) given twice or three daily times daily is recommended where there is a risk or identification of penicillin-resistant *S. pneumoniae.*^27^

Substandard poor-quality or falsified antibiotics promote AMR and the spread of drug-resistant infections.^32^ Since poor-quality antibiotics are unlikely to contain the full dose needed to eliminate all the infecting pathogens this would encourage resistance to develop and allow resistant strains to survive and be transmitted.^30,33^

The quality of medicines, specifically antibiotics, is an important consideration for countries worldwide. The WHO launched a Global Surveillance and Monitoring System (GSMS) for substandard and falsified products.^34^ The GSMS aims to work with WHO member states to improve the quality of reporting of substandard and falsified medical products, and, importantly, to ensure the data collected are analysed and used to influence policy, procedure, and processes to protect public health, at the national, regional and the global level. Use of substandard or falsified antibiotics not only compromises clinical outcome but also risks increased AMR. The most recent summary (2013–17) reported substandard and falsified medicines in 46 member states (including Russia) and antibiotics represent 16.9% of all products reported, second only to malaria drugs (19.6%).^33^

## Local insights

### Clinical microbiologist expert comment

From a clinical microbiology perspective, a steady increase in AMR dictates the need for surveillance to identify resistance trends. AMR monitoring may include a specific healthcare facility or cover multiple participating centres. The first case refers to the local level, and the second, depending on the geographical location of the centres, nationwide or global. The importance of each type of monitoring is undeniable. Local surveillance, however, enables a more rapid development of action plans reacting to locally detected deviations in the aetiological structure and susceptibility profile of pathogens.

Continuous local surveillance may, in a timely way, identify the scope of the local resistance problems, provide data to develop or modify local strategies for the prevention and treatment of infections and provide the background to decrease resistance selection pressure. The situation with AMR has become more critical and significant during the COVID-19 pandemic due to increased inappropriate antimicrobial prescribing.^34,35^ The prevalence of multiresistant Gram-negative bacteria remains high throughout the world, including in inpatients in Russia. According to the AMRmap, there is a universal distribution of nosocomial strains of Enterobacterales producing extended-spectrum β-lactamases and nosocomial carbapenem-resistant *Pseudomonas aeruginosa* and *Acinetobacter baumannii*, limiting options for empirical antimicrobial choice. At the local level, it may be challenging to organize effective interdisciplinary teamwork comprising the clinician, the epidemiologist and the microbiologist. Potentially the impact of the microbiologist’s role on decision making in antimicrobial usage will be supported by introduction of the new clinical specialty of ‘medical microbiologist’ in Russia in 2020. Another potential challenge is sharing reliable surveillance data with the medical community. After development of the AMRmap web platform this issue may be considerably diminished by providing an easy tool to submit up-to-date resistance rates of the most common microorganism, thus enabling in-depth analysis and visualization of AMR data collected from local and national microbiological surveillance programmes in Russia.

### Clinician expert comment

The Russian Federation has developed a system for informing doctors about the level and dynamics of AMR. The system includes professional journals and AMRmap.

An important issue for clinicians is the development of national management guidelines for a range of human diseases. National guidelines have a significant impact on the spread of resistance. The growth of pneumococcal resistance to macrolides, which began in Russia at the end of the 20th century, was partially associated with the positioning of these antibiotics as drugs of choice for the treatment of respiratory infections in many recommendations. Thus, in the 2010 national guidelines for the treatment of CAP, macrolides were positioned as drugs of choice along with β-lactams.

The situation has now improved significantly. In the management guidelines for infectious diseases, there are sections on antibiotic therapy. Recommendations are created by professional organizations based on the principles of evidence-based medicine, taking into account data on resistance, international experience and ensuring that these recommendations are thoroughly reviewed.

Personal experience shows that during recent years, many doctors have improved their professionalism in prescribing antibiotics, but there is no reliable evidence of how accurately the developed recommendations are implemented in practice. The surge in antibiotic consumption during COVID-19 in Russia, in common with the rest of the world, is of concern. As a feature, a sharp increase should be noted in the consumption of not only azithromycin, but also levofloxacin.

## Conclusions

In an era of rising AMR throughout the world, this paper aims to define areas where action is required to tackle AMR by analysing and understanding the current situation within a country or region. Information is presented for Russia concerning antibiotic use and prescribing, approach to AMR, availability of local susceptibility data, use of international and/or local antibiotic prescribing guidelines and how these link to antibiotic availability. To our knowledge this is the first time this information has been reviewed and presented in detail by country.

Until recently, the outpatient use of antibiotics was high, with non-prescription access and self-medication popular in Russia, despite there being a regulation preventing the OTC sale of antibiotics since 2006. In 2017, the sale of antibiotics with a medical prescription was made obligatory and methods were applied to enforce this and attempt to change behaviour and attitudes to the use of antibiotics and to reduce antibiotic resistance. Following global movements to tackle AMR, the Russian government approved a strategic plan in 2017 to prevent and restrict the spread of antibiotic resistance that emphasized the need for public education and surveillance.

In terms of surveillance, there are two large country-wide projects that include respiratory isolates, the AMRmap and PeHASus, which allow access to surveillance data. The view from the clinical microbiologist expert opinion is that there is a steady increase in AMR and this dictates the need for surveillance to identify resistance trends. Several global surveillance studies also include data on isolates from RTIs for Russia including SOAR, ATLAS and SENTRY. The WHO GLASS study will also provide useful data in the future. For the management of the common RTIs, CAP, AOM and ABRS in Russia, clinicians make use of several country-specific local antibiotic prescribing guidelines that need to be updated frequently based on available national/international antibiotic surveillance data. The view of the clinician included within this paper is that development of national management guidelines for a range of human diseases is an important issue for clinicians, since it is felt that national guidelines have a significant impact on the spread of resistance.

A standardized inclusive approach is needed to develop local country-specific guidelines These guidelines would be based on up-to-date surveillance data of isolates from community-acquired infections which would make them more locally relevant for clinicians, reiterating the Consensus Principles as described in the introductory paper to this Supplement.^2^ This would pave the way for improved adherence and a higher level of appropriate antibiotic prescribing in CA-RTIs within Russia which could, in turn, potentially limit AMR development and improve clinical outcomes for patients.
